# The Association of Gum Bleeding with Respiratory Health in a Population Based Study from Northern Europe

**DOI:** 10.1371/journal.pone.0147518

**Published:** 2016-01-25

**Authors:** Francisco Gómez Real, Laura Pérez Barrionuevo, Karl Franklin, Eva Lindberg, Randi Jacobsen Bertelsen, Bryndís Benediktsdóttir, Bertil Forsberg, Thorarinn Gislason, Rain Jögi, Ane Johannessen, Ernst Omenaas, Eirunn Saure, Vivi Schlünssen, Trude Duelien Skorge, Kjell Torén, Antonio Pérez Saavedra, Øistein Svanes, Anne Nordrehaug Åstrøm, Christer Janson, Cecilie Svanes

**Affiliations:** 1 Department of Clinical Science, University of Bergen, Bergen, Norway; 2 Department of Gynecology and Obstetrics, Haukeland University Hospital, Bergen, Norway; 3 Department of Surgical and Perioperative Sciences, Umeå University, Umeå, Sweden; 4 Department of Medical Sciences, Respiratory, Allergy and Sleep Research, Uppsala University, Uppsala, Sweden; 5 University of Iceland, Faculty of Medicine, Reykjavik, Iceland; 6 Department of Public Health and Clinical Medicine, Umeå University, Umeå, Sweden; 7 Lung Clinic, Tartu University Clinics, Tartu, Estonia; 8 Centre for Clinical Research, Haukeland University Hospital, Bergen, Norway; 9 Department of Public Health, Section for Environment, Occupation and Health, Aarhus University, Aarhus, Denmark; 10 Department of Occupational and Environmental Medicine, Sahlgrenska University Hospital, Gothenburg, Sweden; 11 Clinica Dental Pérez Saveedra, Málaga, Spain; 12 Department of Occupational Medicine, Haukeland University Hospital, Bergen, Norway; 13 Department of Clinical Dentistry, University of Bergen, Bergen Norway; Cambridge University, UNITED KINGDOM

## Abstract

**Background:**

There is little knowledge about how oral and respiratory health is interrelated even though the mucosa of the oral cavity and airways constitutes a continuum and the exposures to these are partly similar.

**Aims:**

To investigate whether gum bleeding is related to asthma, respiratory symptoms and self-reported COPD.

**Methods:**

A postal questionnaire including questions about respiratory and oral health was sent to general population samples in seven Northern European centres. In 13,409 responders, gum bleeding when brushing teeth was reported always/often by 4% and sometimes by 20%. Logistic regressions accounted for age, smoking, educational level, centre and gender. Effects of BMI, cardio-metabolic diseases, early life factors, gastro-oesophageal reflux, dental hygiene, nasal congestion, and asthma medication were addressed.

**Results:**

Gum bleeding always/often was significantly associated with ≥3 asthma symptoms (OR 2.58, 95% CI 2.10–3.18), asthma (1.62 [1.23–2.14]) and self-reported COPD (2.02 [1.28–3.18]). There was a dose-response relationship between respiratory outcomes and gum bleeding frequency (≥3 symptoms: gum bleeding sometimes 1.42 [1.25–1.60], often/always 2.58 [2.10–3.18]), and there was no heterogeneity between centres (p_heterogeneity_ = 0.49). None of the investigated risk factors explained the associations. The observed associations were significantly stronger among current smokers (p_interaction_ = 0.004).

**Conclusions:**

A consistent link between gum bleeding and obstructive airways disease was observed, not explained by common risk factors or metabolic factors. We speculate that oral pathogens might have unfavourable impact on the airways, and that the direct continuity of the mucosa of the oral cavity and the airways reflects a pathway that might provide novel opportunities for interventions.

## Introduction

The relationship between oral and respiratory health is not well understood, although the oral cavity and the lower airways are a continuum and exposures are similar, and understanding the relationship might provide novel opportunities for interventions. Periodontitis is a very prevalent chronic inflammatory disease [1] affecting almost 40% of the adult population [2]. The microbial flora of the gingivae is altered and gum bleeding is a key symptom [3, 4]. Periodontitis affects systemic health [5] and is associated with chronic diseases such as diabetes[6], Alzheimer’s disease [7, 8], atherosclerotic vascular diseases [9], rheumatoid arthritis [10], and COPD [11–15]. Gum bleeding, in particular, has been related to poor general health [16]. Aggressive oral pathogens common in periodontitis have systemic effects and have been identified in atheromatous plaques [17], amniotic fluid [18] and placenta [19–21]. Improvement in periodontitis is related to a decrease in systemic inflammation [22] expressed as levels of CRP [23, 24].

Periodontitis has been associated with COPD [11–15] and periodontal treatment was found to result in improved lung function and exacerbation frequency, in a randomized controlled trial of patients with COPD and chronic periodontitis. [25]. It is to our knowledge not known if oral health has a similar effect on asthma, as it seems to have on COPD. There are, however, data suggesting that asthma inhalers and open mouth breathing may lead to decreased saliva production, changes in pH and increased risk of plaques and caries [26–31].

In the present analysis, we investigated the associations between gum bleeding and asthma symptoms, asthma and self-reported COPD. Further, we explored potential explanations for such associations, addressing the role of smoking, asthma medication, metabolic pathways, developmental pathways, and local factors such as dental hygiene and gastro-oesophageal reflux (GERD).

## Methods

### Study population

Respiratory Health in Northern Europe (RHINE) III is the second follow-up of a population-based cohort from seven Northern European centres (Bergen in Norway; Umeå, Gothenburg and Uppsala in Sweden; Aarhus in Denmark; Reykjavik in Iceland and Tartu in Estonia) (www.rhine.nu). The cohort was initially recruited as part of the European Community Respiratory Health Survey (ECRHS) I stage I (www.ecrhs.org). Random population samples of men and women born 1945–73 completed postal questionnaires in 1991–93, 1999–2001 and 2010–12. Sixty-two percent of the original sample responded to RHINE III [32]. The present report presents data from 13,409 persons.

Written consent was obtained from all the participants at each stage of the study. The Regional Committees for Medical and Health Research Ethics West in Norway, the National Bioethics Committee in Iceland, the Research Ethics Committee of the University of Tartu in Estonia, The Regional Ethical Review Board in Uppsala, Sweden and the Scientific Committees for Central Jutland in Denmark approved each stage of the study.

### Respiratory symptoms, asthma and self-reported COPD

The standard ECRHS questions were used to assess respiratory symptoms and diseases (for wording see www.echrs.org). Wheezing and other respiratory symptoms were reported as 12 months prevalence. Current asthma was defined as having had asthma attacks during the last 12 months and/ or currently taking asthma medication. Self-reported COPD was defined by the question “*Has a doctor ever told you that you have chronic obstructive pulmonary disease (COPD)*?” Chronic bronchitis was defined as bringing up phlegm almost every day for at least three months in two consecutive years. “Three or more asthma symptoms” was defined as having answered yes to three or more of the following symptoms in the last 12 months: Wheezing or whistling from the chest; breathless when wheezing; wheezing or whistling when not having a cold; waking with feeling of tightness in chest; having been woken by attack of shortness of breath; having been woken by attack of cough; attack of asthma; currently taking any asthma medicine [33].

### Gum bleeding

The study participants were asked about frequency of gum bleeding: “*Do your gums bleed when you brush your teeth*?”. The response alternatives (with response frequencies) were as follows: always (0.9%), often (3.2%), sometimes (20%), rarely (46%), never (30%).

To support the self-reported assessment of gum bleeding upon tooth brushing, we used measurements of the Community Periodontal Index (CPI) [34] in a sub-sample of 261 persons from Bergen. This sample was investigated as part of the ECRHS III study, performed in Bergen approximately 1 year after the RHINE III questionnaire survey. The CPI index is measured using a standardized protocol for investigation of gum bleeding on probing gingival margins, calculus and pockets. The index is coded from 0 to 4, where 0 denotes none of these periodontal conditions, and 4 denotes the highest score of pathology (pathological pocket of 6 mm or more with or without bleeding and calculus)[35].

### Covariates

Smoking history was assessed by the questions *“Are you a smoker*?*”* and *“Are you an ex-smoker*?*”*; defining never smokers, current smokers and ex-smokers. The study subject’s and parent’s educational level (primary school, secondary/technical education, and college/university) were used as proxy variables for socioeconomic status. Body mass index (BMI) was calculated from self-reported weight and height, as weight in kilos per squared height in meters. Frequency of tooth brushing was assessed by the question “*How often do you usually brush your teeth*?” (Alternatives: Twice daily or more [83%], once daily [15%], less than daily [1.9%]). Poor dental hygiene was defined as brushing teeth less than twice daily. Gastro-oesophageal reflux (GERD) was defined as having heartburn or belching 3–5 night/week or more. Nasal congestion was defines as having a blocked nose >12 weeks during the last 12 months. Cardio-metabolic diseases (hypertension, heart disease, stroke, diabetes, obstructive sleep apnea) were assessed by simple questions of doctor’s diagnosed disease. The following early life variables were assessed: mothers age when giving birth to the participants, parental smoking, severe respiratory infections in childhood and intake of fruit during childhood. The wording of the questions is provided at www.rhine.nu.

### Statistical analyses

Descriptive analyses of the study population by centre and by frequency of gum bleeding were performed. Multiple logistic regressions were used to analyse associations between gum bleeding and respiratory health. Gum bleeding never or rarely was defined as reference category; for interaction and supplementary analyses gum bleeding “sometimes” and “often or always” were collapsed into one category to define gum bleeding; for other analyses “gum bleeding sometimes” and “gum bleeding often or always” was used as separate categories to define increasing levels of gum bleeding. Associations are reported as odd ratios (OR) with 95% confidence intervals (CI).

All analyses were adjusted for sex, age, educational level, smoking habits, and study centre. Analyses were performed with additional adjustments for the following groups of variables: 1) cardio-metabolic factors, 2) factors known to influence oral health (tooth brushing frequency, gastro-oesophageal reflux, nasal congestion), and 3) early life developmental factors. Asthma medication might have a direct effect on the oral mucosa in addition to reflecting asthma disease; analyses with adjustment for asthma medication as well as analyses stratified for asthma medication were therefore presented.

Analysis of interactions between gum bleeding and gender, smoking status, low social class, BMI and asthma medication in effects on asthma symptoms were performed by including the interaction terms in logistic regression models with adjustment for basic characteristics. Potential heterogeneity between centres was analysed using meta-analysis according to derSimonian and Laird[36]. STATA (StataCorp, College Station, TX, USA), version IC 13.1, was used in all the statistical analyses.

## Results

The study population included 53% women aged 34–66 years (mean age 52 years). Gum bleeding rarely or never was reported by 76%, and 83% reported brushing their teeth twice daily. The prevalence of gum bleeding varied considerably between centres, with frequent gum bleeding being reported by 10.3% in Estonia and 2.5% in Umeå. The prevalence of ≥3 asthma symptoms varied between centres from 10% (Arhus) to 19% (Tartu). (Table 1).

**Table 1:** Characteristics of study population by centre, including 13409 participants of the Respiratory Health in Northern Europe study (RHINE) III who provided information about gum bleeding.

	Aarhus	Reykjavik	Bergen	Gothenburg	Umeå	Uppsala	Tartu	All
Numbers *	2340	1910	2338	1692	1917	1914	1298	13409
Gender (% women)	53	54	50	53	53	52	58	53
Mean age (years)	50	53	51	52	53	53	49	52
Current smoking (%)	19	18	24	17	12	11	21	18
Gum bleeding								
- Sometimes (%)	16	16	19	25	22	21	25	20
- Often / Always (%)	3.6	4.2	3.9	3.4	2.5	2.9	10.3	4.1
Poor oral hygiene (brushing teeth once daily or rarely) (%)	11	21	16	15	14	12	43	17
BMI (median, kg/m^2^)	25.2	27.2	25.7	26.0	26.1	25.6	26.7	26.0
Cardio-metabolic disease (%)§	29	45	32	33	36	30	36	34
Gastro-oesophageal reflux (%)	2.2	3.5	2.7	3.6	2.9	3.2	2.5	2.9
Nasal congestion (%)	5.5	10.6	8.3	9.7	10.1	8.1	17.3	9.4
Current asthma medication (%)	5.5	8.3	8.3	7.5	9.6	9.2	3.5	7.6
Wheeze (%)	16	21	21	19	19	17	25	19
≥3 asthma symptoms (%)**	10	16	15	15	15	12	19	14

* Numbers vary slightly between variables due to different frequency of missing

^§^ One of more of the following: Obesity, hypertension, stroke, ischaemic heart disease, diabetes, obstructive sleep apnoea syndrome

** Three or more of the following: Wheezing, breathless when wheezing, wheezing when not having a cold, waking with tightness in chest, waking with shortness of breath, night cough, attack of asthma, current asthma medication.

Gum bleeding was more common in persons with lower educational level. Gum bleeding increased with increasing BMI and was higher in all groups with cardio-metabolic diseases. Gum bleeding was particularly common among persons who brushed their teeth rarely, and in persons with GERD. Childhood respiratory infections and rarely eating fruits in childhood were associated with more gum bleeding in adulthood (Table 2).

**Table 2:** Prevalence of gum bleeding according to population characteristics in 13,409 RHINE III participants.

	% with gum bleeding Sometimes	% with gum bleeding Often/Always	p for difference*
All	20	4.1	
Gender			
Women	20	4.1	0.751
Men	20	4.2	
Age group			
35–44 years	21	4.7	0.242
45–54 years	20	4.2	
55–65 years	20	3.7	
Smoking status			
Never	21	4.1	0.175
Ex	20	4.3	
Current	19	3.9	
Educational level			
Only primary school	24	5.1	<0.001
Intermediate	22	4.4	
University or college	18	3.6	
Cardio-metabolic factors			
BMI			
Underweight (<20 kg/m^2^)	17	3.5	<0.001
Normal weight (20–24.9 kg/m^2^)	18	3.2	
Overweight (25–29.9 kg/m^2^)	21	4.2	
Obese (≥30 kg/m^2^)	24	6.9	
Ischaemic heart disease	26	7.6	<0.001
Stroke	21	8.2	0.011
Sleep apnoea syndrome	26	7.7	<0.001
Diabetes	25	5.6	0.004
Hypertension	23	4.4	0.001
Factors that might directly influence oral health			
Frequency of brushing teeth			
Twice daily	19	3.3	<0.001
Once daily	26	6.4	
Rarely	33	21.5	
Gastro-oesophageal reflux	32	9.7	<0.001
Current nasal congestion †	24	6.2	<0.001
Current asthma medication	22	5.4	0.013
Early life developmental factors			
Mother smoked	20	4.3	0.618
Father smoked	20	4.4	0.074
Severe respiratory infection in childhood	21	5.2	0.048
Mothers age			
<25 years	21	4.1	0.123
25–29 years	20	4.4	
≥30 years	19	3.7	
Eating fruit in childhood			
Seldom/never	21	5.1	0.002
Weekly	21	4.1	
Daily	19	3.6	

* p from chi-square analysis

^†^ Blocked nose >12 weeks last 12 months.

The prevalence of all the respiratory outcomes increased with increasing frequency of gum bleeding. In adjusted analyses, the risk of respiratory symptoms, asthma and self-reported COPD was significantly increased, both among those with gum bleeding sometimes, and among those with gum bleeding often or always, and there was a dose-response relationship in the association for all outcomes. The risk of wheeze with shortness of breath when not having a cold (strongly suggestive of asthma) was almost tripled in those with frequent gum bleeding (Table 3).

**Table 3:** Respiratory symptoms, asthma and self-reported COPD as related to frequency of gum bleeding in a population-based study including between 12803 and 13360 persons with complete data (numbers vary slightly between outcomes due to different frequency of missing).

	Gum bleeding			Gum bleeding			Gum bleeding	
	Never/ rarely			Sometimes			Often / Always	
	%		%	OR (95%CI)*	p-value	%	OR (95%CI)*	p-value
Wheeze	18	ref	22	1.31 (1.17–1.46)	<0.001	32	2.17 (1.78–2.64)	<0.001
Wheeze, with shortness of breath, and when not having a cold	6.5	ref	9.9	1.60 (1.37–1.86)	<0.001	16	2.81 (2.18–3.61)	<0.001
≥3 asthma symptoms†	13	ref	17	1.41 (1.25–1.59)	<0.001	28	2.62 (2.13–3.23)	<0.001
Current asthma medication or attacks	8.0	ref	9.3	1.20 (1.03–1.40)	0.016	11.7	1.71 (1.30–2.25)	<0.001
Chronic bronchitis	11	ref	16	1.47 (1.30–1.66)	<0.001	23	2.35 (1.90–2.91)	<0.001
Self-reported COPD	2.4	ref	3.3	1.60 (1.24–2.08)	<0.001	4.4	1.95 (1.24–3.08)	0.004

* OR from logistic regressions with adjustment for age, gender, smoking status, educational level and study centre.

^†^ Three or more of the following: Wheezing, breathless when wheezing, wheezing when not having a cold, waking with tightness in chest, waking with shortness of breath, night cough, attack of asthma, current asthma medication

With adjustment for different groups of covariates, the associations between gum bleeding and ≥3 asthma symptoms remained strong and statistically significant (Table 4). The association was slightly attenuated by adjustment for cardio-metabolic factors and by adjustment early life developmental factors, while adjustment for dental hygiene, GERD and nasal congestion attenuated the association somewhat more. None of the analysed covariates explained the association of gum bleeding with asthma symptoms (Table 4).

**Table 4:** Association of gum bleeding with ≥3 asthma symptoms*, adjusted for basic characteristics, metabolic factors, early life factors, factors directly affecting oral health, and all of these.

		Gum bleeding Sometimes	Gum bleeding Often / Always
	N	OR (95%CI) †	OR (95%CI) †
Unadjusted	12842	1.42 (1.26–1.60)	2.65 (2.17–3.24)
Adjusted for basic characteristics (smoking, educational level, age, gender, centre)	12814	1.41 (1.25–1.59)	2.62 (2.13–3.23)
Adjusted for basic characteristics and cardio-metabolic factors (BMI, OSAS, ischaemic heart disease, stroke, hypertension, diabetes)	12349	1.34 (1.18–1.52)	2.39 (1.93–2.98)
Adjusted for basic characteristics and factors that may directly influence oral health (tooth brushing frequency, GERD, nasal congestion)	12283	1.24 (1.09–1.41)	2.08 (1.65–2.61)
Adjusted for basic characteristics and asthma medication	12283	1.45 (1.26–1.67)	2.83 (2.23–3.59)
Adjusted for basic characteristics and early life factors (father’s smoking, mother’s smoking, childhood infections, mother’s age, fruit intake in childhood)	10271	1.35 (1.17–1.55)	2.53 (1.98–3.14)
Adjusted for all significant predictors among the above	9789	1.21 (1.01–1.44)	2.11 (1.55–2.86)

* Three or more of the following: Wheezing, breathless when wheezing, wheezing when not having a cold, waking with tightness in chest, waking with shortness of breath, night cough, attack of asthma, current asthma medication

^†^ Odds ratios with 95% confidence intervals, from logistic regression models

The association of gum bleeding (in two categories) with ≥3 asthma symptoms was significant in all investigated strata with the exception of persons using asthma medication (Table 5). The association was significantly stronger among current smokers as compared with never-smokers (p_interaction_ = 0.004), among those with lower socioeconomic status (p_interaction_ = 0.030), and among those not using asthma medication (Table 5).

**Table 5:** The association of gum bleeding (sometimes /often / always *vs* never/ rarely) with ≥3 asthma symptoms, in strata according to gender, smoking status, social class, body mass index and asthma medication.

Variable	Category (n)	OR (95%CI)*	p interaction*
Gender	Men (6045)	1.55 (1.32–1.82)	
	Women (6796)	1.49 (1.28–1.73)	0.513
Smoking status	Never (5845)	1.34 (1.11–1.62)	
	Ex (4773)	1.61 (1.35–1.93)	0.14
	Current (2196)	2.01 (1.61–2.51) §	0.004
Social class†	Higher (12016)	1.54 (1.37–1.73)	
	Lower (787)	2.48 (1.67–3.67)	0.030**
Body mass index	<25 kg/m^2^ (5903)	1.72 (1.43–2.07)	
	25–29.9 kg/m^2^ (4956)	1.43 (1.19–1.71)	0.15
	≥ 30 kg/m^2^ (1911)	1.41 (1.12–1.76)	0.12
Asthma medication	No (11866)	1.75 (1.52–2.00)	
	Yes (908)	1.19 (0.83–1.71)	0.067

* OR from logistic regressions with adjustment for age, gender, smoking status, educational level and study centre (except the variable stratified for). P-value for interaction term from similar model.

^†^ Lower social class: Only primary school in participant and both parents. Higher social class: Any other educational pattern.

^§^ With additional adjustment of number of cigarettes smoked per day OR = 2.04 (1.61–2.59)

** With additional adjustment for tooth brushing frequency p_interaction_ = 0.025

**Fig 1:**
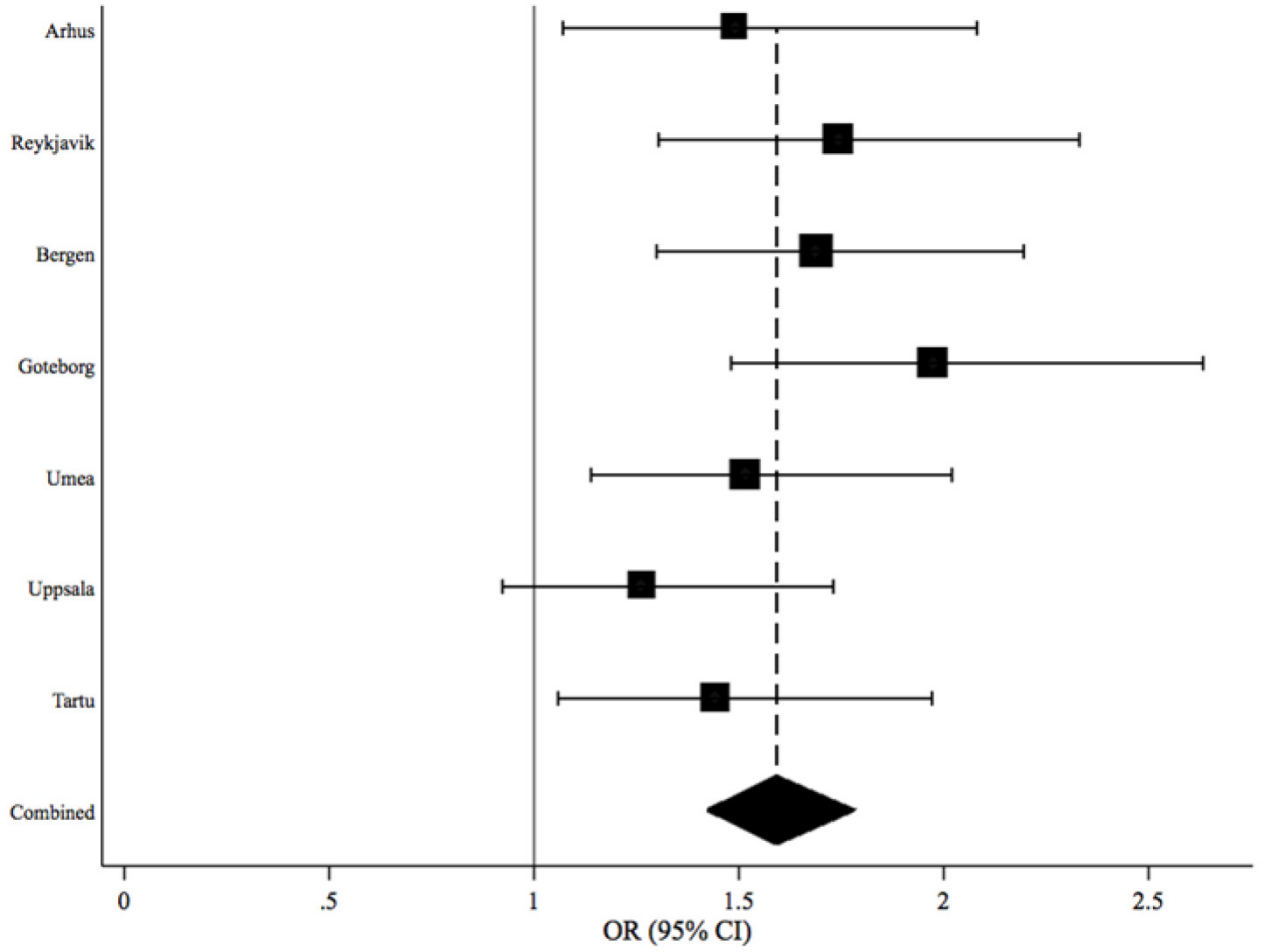
Meta-analysis of the association of ≥3 asthma symptoms with gum bleeding (sometimes/often/always *vs* rarely/never) by study centre, adjusted for age, gender, smoking status and educational level. P_heterogeneity_ = 0.491. Boxes shows odds ratios and horizontal lines show 95% confidence intervals for each study centre, box size is proportional to number of study subjects, diamond shows combined estimate for all centres with 95% confidence interval.

The association of gum bleeding with ≥3 asthma symptoms appeared to be consistent in the different study centres (Fig 1); meta-analysis of this association by centre showed p _heterogeneity_ = 0.491.

Reported increasing frequency of gum bleeding upon tooth brushing was strongly associated with higher degree of periodontal pathology (linear regression, p = 0.003, not altered by adjustment for basic characteristics). CPI index 0 (no periodontal pathology in none of the measured sites in 10 teeth) was present in 71% of those reporting gum bleeding never, 59% of those reporting gum bleeding rarely, and 47% of those reporting gum bleeding sometimes. (Only 11 persons reported gum bleeding always (2) or often (9) in this subsample.)

## Discussion

This analysis of a large population based study found that gum bleeding was significantly associated with asthma symptoms, asthma and self-reported COPD. The associations were consistent across study centres in Northern Europe and present in sub-groups according to gender, smoking habits, social class and body mass index. A clinical validation study in a subsample showed high correlation of self-reported gum bleeding with gum bleeding on probing gingival margins. Adjustment for asthma medication, BMI, cardio-metabolic factors, developmental factors (reflected in early life characteristics) and factors acting locally did not change the association between gum bleeding and asthma symptoms. The association was significantly stronger among current smokers and among persons of lower socioeconomic status, and weaker among persons taking asthma medication.

These findings about asthma are novel, while the observed association of gum bleeding with self-reported COPD is in agreement with previous studies [11, 12]. Effects of asthma inhalers on oral health are described in the literature [26]. However, the present analyses show that asthma symptoms were even more strongly associated with gum bleeding in asthmatics not using asthma medication.

One may envisage several biological mechanisms that could link gum bleeding with asthma. Airborne exposures are likely to be partly common, although filter functions of the nasal cavity reduce exposures to the lower airways. The mucosae of the oral cavity and the upper and the lower airways constitutes a continuum, which suggests a role for oral bacteria in respiratory health [37], i.e. by direct transport of oral bacteria or by transport of inflammatory substances. The present analysis shows that asthma was more strongly associated with gum bleeding among current smokers. Gum bleeding is less common in smokers due to vaso-constrictive effects on the gums. It is known that smoking increases the activity of the aggressive oral pathogen *Porfyromonas Gingivalis* [38–40], and that smokers have characteristic microbial communities that may contribute to respiratory tract complications [41]. Further, smoking destroys the cilia in on the lower airways. Activated aggressive oral pathogens and enhanced deposition along damaged airways mucosa could explain the observed effect modification by smoking, and we hypothesise that oral pathogens and transport from oral to airways mucosae contribute to the association of gum bleeding with respiratory pathology.

Systemic factors are likely to influence the oral mucosa as well as the mucosa of the lower airways. It has been suggested that both periodontal and respiratory diseases are related to common immunological components that affect epithelia in both periodontal and respiratory tissues. One such common factor is matrix metalloproteases [42], responsible for the breakdown of collagen, and found at elevated levels during the periodontal breakdown process as well as related to bronchial remodelling in individuals with severe asthma[43, 44]. In addition, gingival IgE concentrations have been found to be elevated both among patients with asthma and among patients with periodontitis [45]. Individual variations in defence mechanisms in the oral cavity and the airways might have similarities, and this could possibly contribute to the association of asthma with gum bleeding.

One might expect that systemic inflammation would be important for the relationship between periodontal health and obstructive airways disease [46]. In the present analysis this was not found, as adjustments for a range of cardio-metabolic factors characterised by systemic inflammation did not attenuate the association of asthma with gum bleeding, and the association was present in all sub-groups when stratifying for BMI. One may therefore suspect that other mechanisms than common systemic inflammation are important for the association between gum bleeding and obstructive airways disease.

GERD causes acidic exposure to both the oral cavity and the airways, and it is well known that GERD is a cause of gingivitis and gum bleeding [47] as well as of asthma and respiratory symptoms [48]. Adjustment for GERD attenuated slightly the association of gum bleeding with asthma. The findings suggest that factors acting on the mucosae of the oral cavity and the airways may be of importance for the link between oral and airways health.

### Methodological considerations

Self-reported gum bleeding may be subject to reporting bias. We measured CPI index in a subsample of participants in one centre (Bergen, Norway), and found that self-reported gum bleeding was strongly related to measured periodontal pathology. Thus, the report of gum bleeding appeared to reflect periodontal pathology reasonably well. However, any errors in reporting of gum bleeding are unlikely to be differential according to asthma status, thus, such error is likely to have caused non-differential bias and attenuated true associations.

Smokers experience generally less gum bleeding than non-smokers. Rest confounding by smoking would thus be negative, and would have attenuated rather than increased observed associations between asthma symptoms and gum bleeding. Accordingly, adjustment for number of cigarettes smoked daily among current smokers increased very slightly the association of gum bleeding with asthma symptoms. We also found that the association between gum bleeding was particularly strong in current smokers.

## Conclusions

The present analysis suggests an important relationship between oral health and asthma. The findings were consistent by various ways of analyses, consistent between centres with different prevalence of gum bleeding and asthma, and not related to effects of asthma medication. Interpreted in the light of previous literature, the findings suggest that aggressive oral pathogens as well as local factors of the mucosa of the oral cavity and airways may be important for the link between oral and airways health. Further insight into this might provide important opportunities for intervention that have been very little explored until now.

## Abbreviations

BMI: body mass index
CI: confidence interval
COPD: Chronic obstructive lung disease
CPI: Community Periodontal Index
ECRHS: European Respiratory Health Survey
GERD: gastroesophageal reflux disease
IgE: immunoglobulin E
OR: odds ratio
RHINE: Respiratory Health in Northern Europe
ECRHS: European Community Respiratory Health Survey
